# Regulatory Effects of Mepiquat Chloride on Root–Shoot Biomass Accumulation and Physiological Homeostasis in Different Soybean Varieties Under Drought Stress

**DOI:** 10.3390/plants15132031

**Published:** 2026-06-30

**Authors:** Xinyu Zhou, Xiyue Wang, Wei Zhao, Yuanqi Ma, Shoukun Dong

**Affiliations:** College of Agriculture, Northeast Agricultural University, Harbin 150030, China

**Keywords:** soybean, mepiquat chloride, drought stress, root–shoot allometry, antioxidant defense, osmotic adjustment

## Abstract

Drought is one of the major abiotic stresses limiting soybean production, and its detrimental effects are jointly influenced by stress intensity, duration, and cultivation conditions. To investigate the morphological and physiological regulatory mechanisms by which mepiquat chloride (DPC) alleviates drought stress at the soybean seedling stage, this study used the drought-tolerant soybean cultivar Heinong 44 (H-44) and the drought-sensitive cultivar Heinong 65 (H-65) as experimental materials. Osmotic stress was simulated with 10% PEG-6000 at the V2 stage, and the effects of foliar application of different DPC concentrations (125–500 mg/L) on soybean morphology, biomass allocation, antioxidant systems, and osmotic adjustment capacity were systematically analyzed. The results showed that drought stress significantly inhibited the growth of both soybean cultivars and induced severe oxidative damage. Appropriate DPC concentrations moderately restricted shoot growth to reduce transpiration area while promoting root growth to enhance water acquisition capacity. The optimal DPC concentrations for alleviating drought stress were 200 mg/L for H-44 and 275 mg/L for H-65. Allometric growth analysis indicated that drought disrupted the original root–shoot growth pattern, whereas appropriate DPC concentrations significantly promoted dry matter accumulation in drought-stressed plants and improved root–shoot growth coordination. However, an excessive concentration of DPC (500 mg/L) caused an abnormal deviation in the growth trajectory. In addition, appropriate DPC concentrations synergistically enhanced the activities of superoxide dismutase (SOD), peroxidase (POD), catalase (CAT), and ascorbate peroxidase (APX) in leaves and roots under drought conditions; promoted the accumulation of proline (Pro), soluble sugars (Ss), and soluble proteins (Sp); effectively reduced the contents of malondialdehyde (MDA) and hydrogen peroxide (H_2_O_2_); and protected cell membrane stability. In conclusion, DPC synergistically enhances drought resistance in soybean by reshaping the root–shoot allometric growth configuration and systematically activating physiological defense networks, providing a theoretical basis for chemically regulated cultivation of soybean under stress conditions.

## 1. Introduction

Soybean (*Glycine max* [L.] Merr.) is a globally important crop used for grain, oil, and feed. It not only provides high-quality plant protein and edible oil for human consumption but also serves as an indispensable feed source for animal husbandry [1]. In recent years, with the continuous growth in demand from the feed processing, edible oil, and soybean product industries in China, soybean consumption has remained at a high level, whereas domestic production has been insufficient to fully meet demand, resulting in a high dependence on imports. In 2024, China imported approximately 105 million tons of soybeans, while domestic production was approximately 20.65 million tons, meaning that imports were nearly five times domestic output [2]. Therefore, improving soybean yield and yield stability in China is of great significance for ensuring national grain and oil security, optimizing the supply structure, and reducing dependence on imports [3]. Heilongjiang Province is the most important soybean-producing region in China, contributing more than 40% of national soybean production, and thus plays a crucial role in stabilizing domestic soybean production and safeguarding national grain and oil security. However, with the intensification of global climate change, drought has become one of the major abiotic stresses limiting soybean growth, development, and yield improvement [4].

The effects of drought on soybean are highly stage-dependent, and the extent of damage is jointly determined by stress intensity, duration, cultivar, growth stage, and cultivation environment [5,6,7]. Drought stress not only causes plant stunting and leaf area reduction but also disrupts the metabolic balance of reactive oxygen species (ROS), thereby inducing membrane lipid peroxidation damage [8]. In addition, drought alters the biomass allocation pattern between shoots and roots, affecting root water uptake capacity and canopy establishment, and consequently weakening overall plant growth [9]. Previous studies have shown that drought stress occurring at any soybean growth stage can severely impair yield components and ultimately lead to significant yield losses of 21–63% [10,11]. Among the different growth stages, the seedling stage is critical for root establishment, leaf area expansion, and canopy structure formation. Water deficit during this stage can directly affect subsequent reproductive growth and yield potential. It has been reported that seedling-stage drought can reduce shoot dry weight by 4.44–65.94% in soybean genotypes with different drought tolerance levels and decrease net photosynthetic rate, stomatal conductance, and transpiration rate by 60–80% [12]. Therefore, investigating drought-resistance regulation at the seedling stage is of great importance for improving early stress tolerance and stabilizing soybean yield potential.

Chemical regulation using exogenous plant growth regulators offers a highly cost-effective approach to agronomic management [13]. Among these, mepiquat chloride (DPC), a well-known systemic growth retardant, has been extensively adopted to modulate canopy structure and stress-resistant cultivation in crops such as cotton [14,15]. Previous studies have indicated that optimal concentrations of DPC can inhibit gibberellin biosynthesis and delay cell elongation, thereby shaping a compact plant morphology. Simultaneously, DPC can enhance photosynthetic efficiency, promote root development, and improve plant tolerance to adverse conditions such as drought and salinity [16,17,18]. As the primary organ for sensing soil water deficit, the morphological development and physiological state of roots play a decisive role in maintaining water uptake and mitigating drought-induced damage [19]. Under drought stress, plants frequently adapt to harsh environments by adjusting their dry matter allocation strategies [20]. However, current research regarding the application of DPC in soybean predominantly focuses on single plant organs, lacking a systematic analysis that treats the shoot and root as a unified whole. In fact, plant drought response is a highly coordinated, multi-organ systemic process. The regulatory mechanisms concerning how DPC modulates biomass allocation in different drought-resistant soybean genotypes at the whole-plant level, and how it synergizes antioxidant enzymes and osmotic adjustment substances in both roots and leaves to maintain overall plant homeostasis, remain largely obscure. Therefore, this study was designed not only to verify whether DPC can enhance soybean drought tolerance, but also to clarify the systemic regulatory mechanism by which DPC coordinates root–shoot biomass allocation and root–leaf physiological defense responses under drought stress.

Based on this background, the present study used the drought-tolerant soybean cultivar Heinong 44 (H-44) and the drought-sensitive soybean cultivar Heinong 65 (H-65) as experimental materials. Their contrasting drought tolerance was classified based on previous drought-resistance evaluations at the germination and/or seedling stages [21]. PEG-6000 was used to simulate drought stress, and the effects of foliar application of different DPC concentrations on soybean morphological development, biomass allocation, antioxidant metabolism, and osmotic adjustment capacity were systematically investigated. By integrating root–shoot allometric growth models, correlation analysis, and principal component analysis (PCA), this study aimed to elucidate the physiological mechanisms by which DPC alleviates drought stress in soybean and to provide a theoretical basis for the development and application of chemically regulated drought-resistant cultivation practices.

## 2. Results

### 2.1. Effects of DPC Application on the Shoot Morphology of Soybean Under Drought Stress

**Figure 1:**
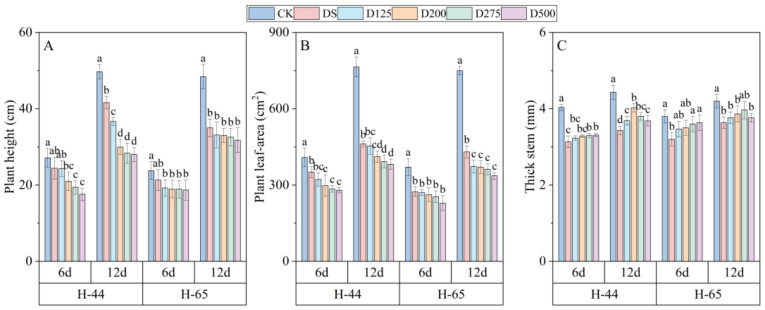
Effects of DPC application on soybean shoot morphology under drought stress. (**A**) Plant height; (**B**) plant leaf area; (**C**) stem thickness. Data are presented as the mean ± SD of three replicates. Different lowercase letters indicate significant differences among treatments within the same cultivar at the same sampling time (*p* < 0.05, Duncan’s multiple range test). H-44 and H-65 represent Heinong 44 and Heinong 65, respectively. The 6 d and 12 d symbols indicate the number of days after treatment. CK represents the control, DS represents drought stress, and D125, D200, D275, and D500 represent foliar application of DPC at concentrations of 125, 200, 275, and 500 mg/L under drought stress, respectively. The same definitions apply to the following figures.

Drought stress significantly inhibited the increase in plant height of both soybean cultivars (Figure 1A), and this inhibitory effect intensified with prolonged stress duration. At 12 days of treatment, the plant heights of H-44 and H-65 under drought stress (DS) decreased by 16.34% and 27.68%, respectively, compared with the CK. Under drought conditions, foliar application of DPC further reduced soybean plant height, mainly reflecting the regulatory effect of DPC on stem elongation. The most pronounced reduction was observed under the D500 treatment. At 12 days, the plant heights of H-44 and H-65 under D500 decreased by 32.65% and 9.27%, respectively, compared with the DS group. The variation trend of leaf area was consistent with that of plant height (Figure 1B). Drought stress significantly hindered leaf expansion in both cultivars. At 12 days of treatment, the leaf areas of H-44 and H-65 under DS decreased by 39.59% and 42.61%, respectively, compared with the CK. After DPC application, the restriction of leaf area increased continuously with increasing DPC concentration, although the differences among the medium- and high-concentration treatments (D200–D500) gradually became less pronounced. Under the D500 treatment, the leaf areas of H-44 and H-65 decreased by 50.04% and 55.14%, respectively, compared with the DS group. For stem diameter (Figure 1C), drought stress significantly reduced the stem diameter of both cultivars, whereas DPC application alleviated this reduction to some extent, with different response patterns observed at 6 and 12 days of stress. At 6 days of stress, stem diameter in both cultivars increased gradually with increasing DPC concentration and reached the maximum under the D500 treatment. Under this treatment, the stem diameters of H-44 and H-65 increased by 17.98% and 4.39%, respectively, compared with the DS group. However, at 12 days of stress, stem diameter in both cultivars first increased and then decreased with increasing DPC concentration. The maximum values for H-44 and H-65 were observed under the D200 and D275 treatments, respectively, representing increases of 17.15% and 9.18% compared with the DS group.

### 2.2. Effects of DPC Application on Soybean Root Morphology Under Drought Stress

**Figure 2:**
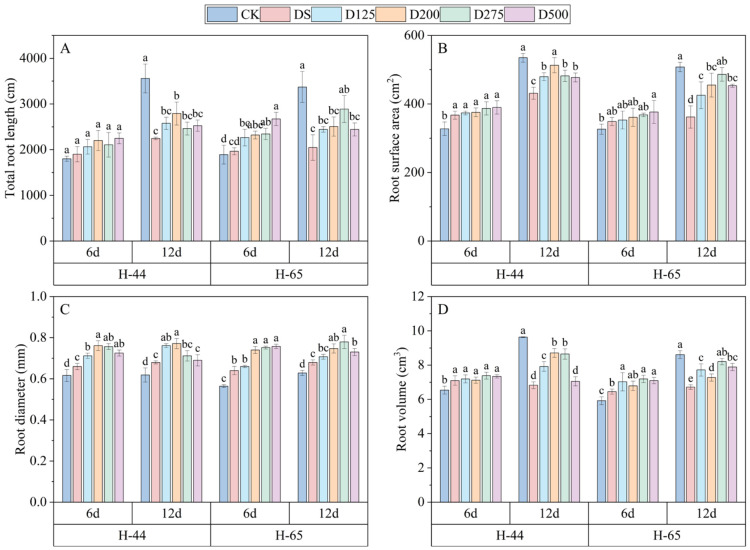
Effects of DPC application on soybean root morphology under drought stress. (**A**) Total root length; (**B**) Root surface area; (**C**) Root diameter; (**D**) Root Volume. Different lowercase letters indicate significant differences among treatments within the same cultivar at the same sampling time (*p* < 0.05, Duncan’s multiple range test).

Foliar spraying of DPC under drought stress conditions prominently modulated the growth of soybean roots (Figure 2). After 6 days of drought exposure, the DS treatment showed a slight promotive effect on both root surface area and total root length across both cultivars. However, when the drought period was extended to 12 days, it severely restricted root elongation and expansion. Specifically, at day 12, the DS group exhibited reductions of 36.93% (H-44) and 39.31% (H-65) in total root length, and 19.34% (H-44) and 28.73% (H-65) in root surface area relative to the control (CK). Notably, DPC application effectively mitigated the repressive effects of drought on root lengthening. For both cultivars, both root surface area and total root length exhibited a trend of initially increasing and then declining with rising DPC concentrations. Of all treatments, the D200 concentration yielded the most prominent recovery in H-44, augmenting its total root length and root surface area by 24.30% and 18.93%, respectively, in comparison with the DS group.

The ameliorating role of DPC in root system morphology under drought stress was further supported by changes in root volume and diameter. On day 12, drought stress led to a 29.18% (H-44) and 22.07% (H-65) decline in root volume compared to CK, whereas root diameter rose by 9.90% and 8.16%, respectively. With DPC treatment, root volume under drought conditions was substantially restored, accompanied by a further increment in root diameter. Under D200 application, H-44 reached its peak root volume and diameter, which were 27.71% and 13.40% higher than those of the DS control, respectively. For H-65, the optimal performance was recorded under the D275 treatment, showing respective increases of 22.15% and 14.71% relative to the DS group.

### 2.3. Effects of DPC Application on Dry Matter Accumulation in Different Soybean Organs Under Drought Stress

**Figure 3:**
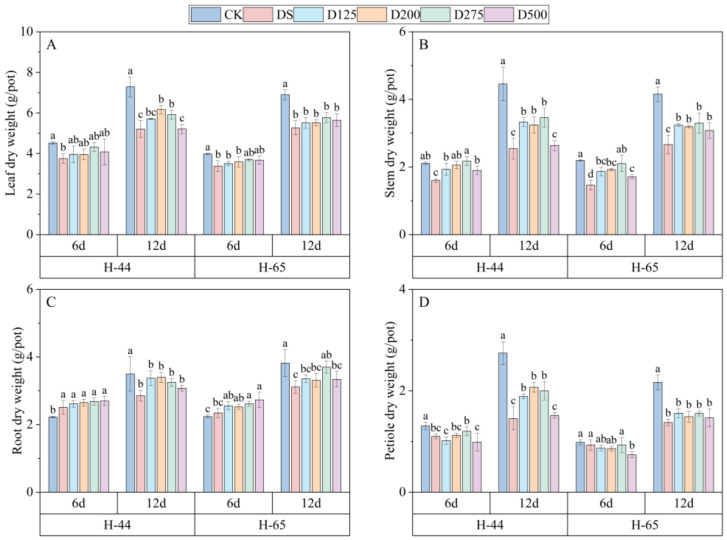
Effects of DPC application on dry weights of different soybean organs under drought stress. (**A**) Leaf dry weight; (**B**) stem dry weight; (**C**) root dry weight; (**D**) petiole dry weight. Different lowercase letters indicate significant differences among treatments within the same cultivar at the same sampling time (*p* < 0.05, Duncan’s multiple range test).

Drought stress significantly inhibited biomass accumulation in both the aboveground and belowground parts of soybean plants (Figure 3). At 12 d of treatment, the leaf dry weights of H-44 and H-65 under drought stress decreased by 28.28% and 23.62%, respectively, compared with CK, while stem dry weights decreased by 42.98% and 35.88%, respectively. DPC application exhibited a marked compensatory effect on biomass accumulation under drought stress, and this effect varied among DPC concentrations. For H-44, the D200 treatment showed the most pronounced alleviating effect. At 12 d of treatment, the dry weights of leaves, stems, roots, and petioles increased by 17.91%, 31.06%, 18.45%, and 37.66%, respectively, compared with DS. For H-65, D275 showed a stronger compensatory effect; at 12 d of treatment, the dry weights of leaves, stems, and roots increased by 9.59%, 25.39%, and 16.54%, respectively, compared with DS, whereas no significant difference was observed in petiole dry weight.

The responses of dry matter accumulation in different organs to DPC application differed between 6 d and 12 d of stress. At 6 d of stress, DPC application exerted a particularly prominent promoting effect on root dry weight. Root dry weight in both varieties increased significantly with increasing DPC concentration, with H-44 and H-65 showing increases of 20.45% and 17.29%, respectively, under the D500 treatment compared with DS. However, as the stress duration was extended to 12 d, the compensatory effect of DPC on aboveground dry matter accumulation became more evident. Stem dry weight in both varieties showed a significant recovery, and petiole dry weight in H-44 was also markedly restored.

### 2.4. Effects of DPC Application on Biomass Allocation Proportions of Soybean Under Drought Stress

**Figure 4:**
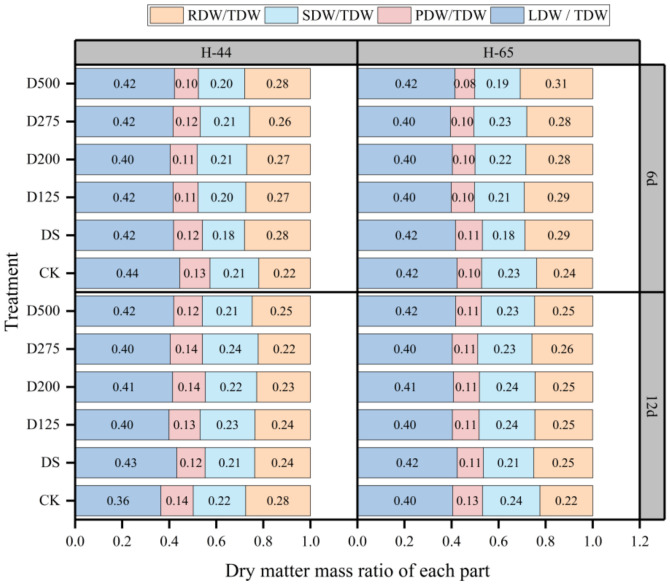
Effects of DPC application on biomass allocation proportions of soybean under drought stress. RDW/TDW: Root dry weight/Total dry weight; SDW/TDW: Stem dry weight/Total dry weight; PDW/TDW: Petiole dry weight/Total dry weight; LDW/TDW: Leaf dry weight/Total dry weight.

Drought stress significantly altered the dry matter allocation proportions among different soybean organs (Figure 4), and the allocation patterns exhibited obvious differences between 6 d and 12 d of treatment. At 6 d of stress, both varieties exhibited an adaptive strategy by increasing belowground investment. The DS treatment prompted both varieties to transfer more dry matter to the roots; the root-to-total dry weight ratio (RDW/TDW) of H-44 and H-65 increased by 27.80% and 21.03%, respectively, compared with the CK, while the stem-to-total dry weight ratio (SDW/TDW) decreased by 13.93% and 22.73%, respectively. Foliar application of DPC under drought conditions effectively alleviated the excessive inhibition of aboveground growth while maintaining a relatively high proportion of root dry matter allocation. Specifically, the SDW/TDW of H-44 under the D200 treatment and H-65 under the D275 treatment significantly rebounded by 17.61% and 24.74%, respectively, compared with the DS group.

As the stress duration extended to 12 d, the allocation strategies of the two varieties under drought diverged. In H-44, the RDW/TDW and SDW/TDW under the DS treatment decreased by 13.92% and 5.47%, respectively, compared with the CK. Conversely, H-65 still needed to maintain a higher belowground investment to cope with the continuous water deficit, resulting in an 11.82% increase in RDW/TDW and a 12.05% decrease in SDW/TDW compared with the CK. DPC application significantly altered the dry matter allocation patterns of both varieties, and this regulatory effect was genotype-dependent. Under the D275 treatment, the SDW/TDW and petiole-to-total dry weight ratio (PDW/TDW) of H-44 increased by 12.20% and 13.27%, respectively, compared with the DS treatment, thereby promoting dry matter transfer to the aboveground parts. In H-65, the D275 treatment increased the SDW/TDW and RDW/TDW by 7.36% and 3.03%, respectively, compared with the DS group.

### 2.5. Effects of DPC Application on the Root–Shoot Growth Relationship of Soybean Under Drought Stress

**Figure 5:**
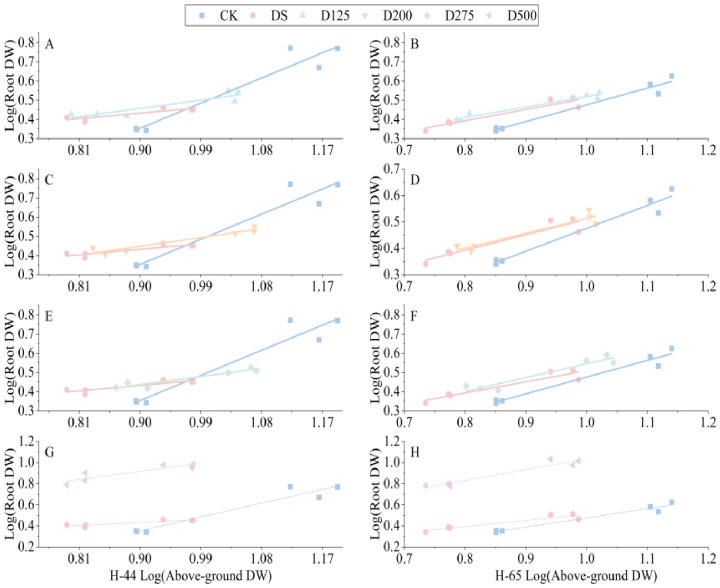
Effects of DPC application on the root–shoot allometric growth relationship of soybean under drought stress. (**A**,**C**,**E**,**G**) Relationships between root dry weight and above-ground dry weight in H-44 from CK to D500; (**B**,**D**,**F**,**H**) relationships between root dry weight and above-ground dry weight in H-65 from CK to D500. Both the x- and y-axes were log_10_-transformed. Scatter points with different colors or shapes represent different experimental treatments: CK, control treatment; DS, drought stress treatment; and D125, D200, D275, and D500, foliar application of DPC at concentrations of 125, 200, 275, and 500 mg/L under drought stress, respectively. Solid lines indicate the fitted linear regression lines based on the log_10_-transformed data.

Root–shoot allometric growth analysis further revealed the biomass allocation characteristics of soybean varieties with different drought tolerances and their differential responses to DPC application (Figure 5 and Table 1). Under normal water conditions (CK), the allometric scaling exponent of H-44 (b = 1.45) was higher than that of H-65 (b = 0.87), indicating that H-44 had a stronger tendency toward belowground biomass investment. Drought stress disrupted the original root–shoot allometric balance in both varieties. Compared with CK, the allometric slopes of H-44 and H-65 under the DS treatment decreased to 0.32 and 0.58, respectively, indicating that drought stress severely disturbed the original coordinated growth pattern between roots and shoots and reduced root–shoot growth coordination.

After DPC application under drought stress, the allometric growth trajectories of both varieties changed markedly, and excessive regulation was observed under the D500 treatment. In H-44, the allometric slopes under the D125–D275 treatments remained relatively stable, ranging from 0.47 to 0.51, suggesting that appropriate DPC concentrations could help maintain drought-adaptive growth homeostasis to some extent. In addition, the coefficient of determination (R^2^) for the root–shoot growth relationship of H-44 decreased to 0.81 under drought stress, indicating that drought disrupted the intrinsic proportional relationship between root and shoot growth and led to growth decoupling. However, after the application of an appropriate DPC concentration, such as D200, the allometric slope b remained stable at 0.51, while R^2^ increased to 0.92, with a Pearson’s correlation coefficient of 0.96. This indicated that a strong linear relationship between root and shoot biomass was re-established. The convergence of the b value toward approximately 0.5 may represent an adaptive allocation pattern reconstructed through active trade-offs between root and shoot growth under drought conditions. However, when the DPC concentration increased to D500, the slope of H-44 increased to 0.84, which may be attributed to excessive inhibition of shoot growth caused by high DPC concentration, thereby forcing a shift in dry matter allocation. For H-65, the D125 and D200 treatments did not markedly alter the allometric growth trajectory of drought-stressed plants. In contrast, the D275 treatment increased the slope to 0.72, indicating a favorable regulatory effect. However, under the D500 treatment, the allometric slope of H-65 further increased to 1.02, even exceeding the CK level. This suggests that an excessively high DPC concentration may severely inhibit shoot growth, resulting in an abnormal root–shoot allometric relationship.

### 2.6. Effects of DPC Application on MDA and H_2_O_2_ Contents in Soybean Under Drought Stress

**Figure 6:**
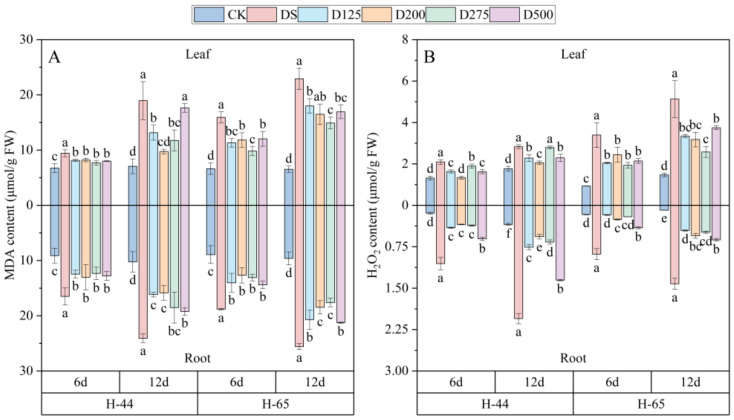
Effects of DPC application on MDA and H_2_O_2_ contents in soybean under drought stress. (**A**) MDA content in leaves and roots; (**B**) H_2_O_2_ content in leaves and roots. Different lowercase letters indicate significant differences among treatments within the same cultivar at the same sampling time (*p* < 0.05, Duncan’s multiple range test).

MDA and H_2_O_2_ contents are key indicators for evaluating the degree of cell membrane lipid peroxidation and ROS metabolism disorders under drought stress (Figure 6). The results showed that drought stress significantly increased the MDA and H_2_O_2_ contents in both the leaves and roots of the two soybean varieties at 6 d and 12 d of treatment. These contents continued to accumulate with prolonged stress duration, peaking at 12 d. Foliar application of DPC significantly alleviated the oxidative stress induced by drought, but its alleviating effects exhibited obvious concentration- and genotype-dependent differences. At 12 d of treatment, the leaf MDA and H_2_O_2_ contents of H-44 under the D200 treatment significantly decreased by 48.62% and 27.58%, respectively, compared with the DS group. In contrast, H-65 required a higher DPC concentration for an optimal response; the greatest reduction in oxidative damage was observed under the D275 treatment, where MDA and H_2_O_2_ contents decreased by 34.92% and 49.77%, respectively, compared with the DS group. However, when the DPC concentration increased to D500, the MDA and H_2_O_2_ contents of both varieties exhibited a clear increasing trend, indicating that an excessively high DPC concentration could weaken its protective effect on the plants and might even trigger secondary stress.

Furthermore, the physiological response patterns of roots and leaves were similar but exhibited certain differences: roots suffered more severe oxidative damage from drought but were also more sensitive to DPC regulation. At 12 d, the D200 treatment decreased the MDA and H_2_O_2_ contents in the roots of H-44 by 34.17% and 77.53%, respectively, compared with the DS group, whereas the D275 treatment decreased those in H-65 by 31.23% and 65.75%, respectively. These findings indicate that an appropriate DPC concentration can effectively scavenge H_2_O_2_ and reduce membrane lipid peroxidation, thereby maintaining cell membrane stability in soybean plants under drought stress. Conversely, an excessively high concentration (D500) carries the risk of exacerbating oxidative damage.

### 2.7. Effects of DPC Application on Antioxidant Enzyme Activities in Soybean Under Drought Stress

**Figure 7:**
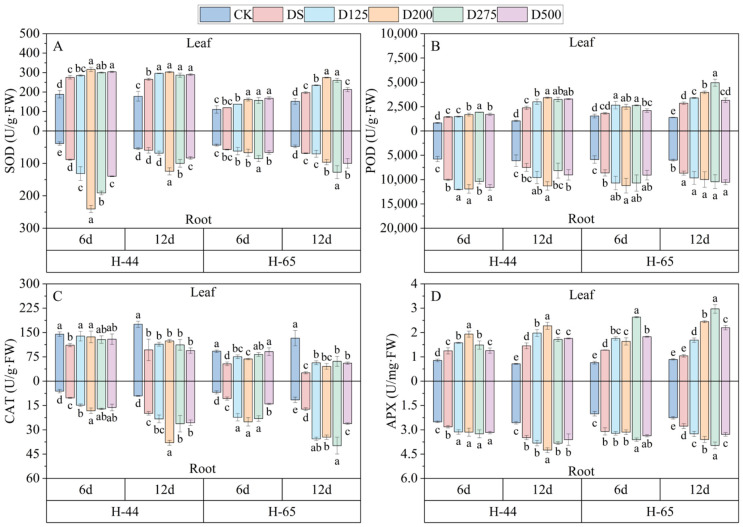
Effects of DPC application on antioxidant enzyme activities in soybean under drought stress. (**A**) SOD activity in leaves and roots; (**B**) POD activity in leaves and roots; (**C**) CAT activity in leaves and roots; (**D**) APX activity in leaves and roots. Different lowercase letters indicate significant differences among treatments within the same cultivar at the same sampling time (*p* < 0.05, Duncan’s multiple range test).

As shown in Figure 7, drought stress significantly affected antioxidant enzyme activities in soybean leaves and roots, with distinct responses between different organs. At 6 d of stress, the DS treatment induced increases in the activities of SOD, POD, and APX in leaves, whereas CAT activity decreased significantly. In contrast to leaves, CAT activity in roots increased significantly at 6 d of stress and was not markedly inhibited. As the stress duration was extended to 12 d, this organ-specific response remained evident.

Foliar application of DPC significantly enhanced antioxidant enzyme activities in both soybean varieties under drought stress. At 12 d of treatment, the activities of SOD, POD, APX, and CAT in leaves of H-44 under the D200 treatment increased by 14.05%, 43.32%, 56.63%, and 28.77%, respectively, compared with the DS treatment, effectively alleviating the drought-induced inhibition of leaf CAT activity. For H-65, the D275 treatment increased leaf SOD, POD, CAT, and APX activities by 31.99%, 73.49%, 137.49%, and 184.26%, respectively, compared with DS, indicating a pronounced synergistic enhancement effect. In addition, antioxidant enzyme activities generally declined under the D500 treatment, which was consistent with the accumulation trends of MDA and H_2_O_2_.

In roots, DPC also showed a significant enhancing effect on antioxidant enzyme activities. At 12 d of treatment, D200 increased the activities of SOD, POD, CAT, and APX in H-44 roots by 108.64%, 50.67%, 91.68%, and 22.14%, respectively, compared with DS. Similarly, D275 increased the activities of these four enzymes in H-65 roots by 85.03%, 20.43%, 130.00%, and 42.85%, respectively. These results indicate that, under drought stress, an appropriate concentration of DPC can enhance the reactive oxygen species-scavenging capacity of soybean plants by inducing the coordinated upregulation of antioxidant enzyme activities, thereby maintaining cellular redox homeostasis.

### 2.8. Effects of DPC Application on Osmotic Adjustment Substances in Soybean Under Drought Stress

**Figure 8:**
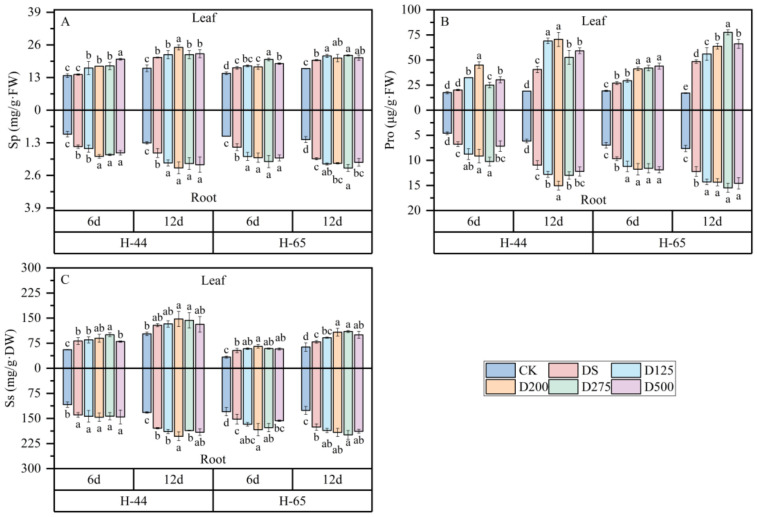
Effects of DPC application on osmotic adjustment substances in soybean under drought stress. (**A**) SP content in leaves and roots; (**B**) Pro content in leaves and roots; (**C**) SS content in leaves and roots. Different lowercase letters indicate significant differences among treatments within the same cultivar at the same sampling time (*p* < 0.05, Duncan’s multiple range test).

As shown in Figure 8, drought stress significantly increased the contents of Proline (Pro), soluble sugar (Ss), and soluble protein (Sp) in soybean leaves and roots, and these contents continued to accumulate with prolonged stress duration. At 12 d of treatment, the Pro contents in the leaves of H-44 and H-65 under the DS treatment increased by 113.61% and 184.91%, respectively, compared with CK. The Ss contents increased by 25.14% and 23.94%, and the Sp contents increased by 25.78% and 20.10%, respectively. Simultaneously, the Pro, Ss, and Sp contents in the roots of H-44 and H-65 were significantly elevated by 77.81% and 59.65%, 35.60% and 39.72%, and 31.00% and 65.04%, respectively, compared with CK.

Foliar application of DPC further enhanced the accumulation of these osmotic adjustment substances, exhibiting obvious genotype- and concentration-dependent differences. In leaves, at 12 d of treatment, the Pro, Ss, and Sp contents of H-44 under the D200 treatment increased by 73.08%, 14.32%, and 19.14%, respectively, compared with DS. For H-65, the D275 treatment increased these three indicators by 60.14%, 39.63%, and 9.61%, respectively, compared with DS. As the direct perception organs for drought stress, roots exhibited a more active regulatory response to DPC application. At 12 d, the D200 treatment increased the Pro, Ss, and Sp contents in H-44 roots by 44.59%, 54.06%, and 61.43%, respectively, compared with DS. Similarly, the D275 treatment increased the Pro, Ss, and Sp contents in H-65 roots by 25.56%, 7.01%, and 98.17%, respectively, compared with DS. Furthermore, when the DPC concentration was increased to D500, the contents of these osmotic adjustment substances in both leaves and roots declined compared with the appropriate concentrations (D200 and D275). This indicates that an excessively high DPC concentration may interfere with the normal metabolic processes of the plants, thereby weakening their osmotic adjustment capacity.

### 2.9. Correlation Analysis of Physiological Indicators in Soybean Under Drought Stress and DPC Application

**Figure 9:**
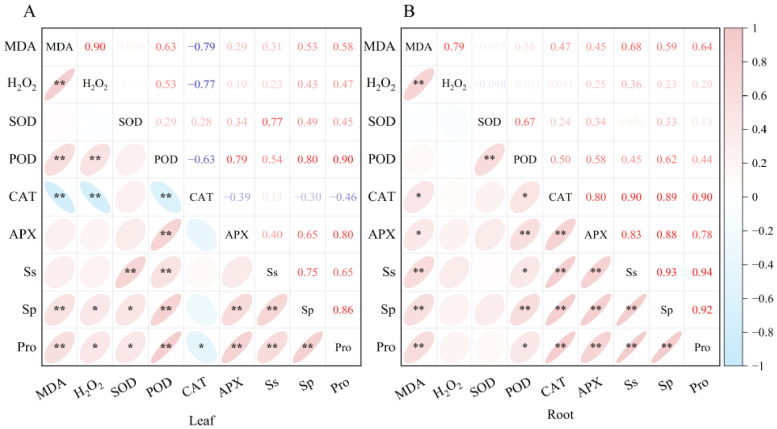
Effects of DPC application on correlations among physiological indicators in soybean leaves and roots under drought stress. (**A**) represents leaves, and (**B**) represents roots. The color scale on the right indicates the magnitude of Pearson correlation coefficients, where red and blue hues designate positive and negative associations, respectively. Darker colors indicate stronger correlations. The values within the squares represent Pearson correlation coefficients. Asterisks denote statistical significance thresholds (* *p* ≤ 0.05; ** *p* ≤ 0.01).

To further clarify the physiological mechanisms by which DPC regulates drought tolerance in soybean, correlation analysis was performed on physiological parameters in leaves and roots (Figure 9). The results showed that the physiological responses of soybean leaves and roots exhibited both coordinated regulatory characteristics and certain organ specificity. In leaves, POD was significantly or highly significantly positively correlated with Ss, Sp, and Pro, with correlation coefficients of 0.54, 0.80, and 0.90, respectively. SOD also showed a strong positive correlation with Ss, with a correlation coefficient of 0.77. These results indicate that the enhancement of antioxidant enzyme activities in leaves was closely associated with the accumulation of soluble sugar, soluble protein, and proline. In contrast, CAT was negatively correlated with MDA, H_2_O_2_, POD, Sp, and Pro, among which the correlations between CAT and MDA, H_2_O_2_, and POD reached significant or highly significant levels. This suggests that CAT in leaves may exhibit regulatory characteristics different from those of POD and SOD.

In roots, APX and CAT showed stronger positive correlations with osmotic adjustment substances. Specifically, the correlation coefficients of APX with Ss, Sp, and Pro were 0.83, 0.88, and 0.78, respectively, while those of CAT with Ss, Sp, and Pro were 0.80, 0.90, and 0.90, respectively; all of these correlations reached significant or highly significant levels. These findings indicate that APX and CAT may be key enzymes involved in DPC-regulated antioxidant defense in roots and may act synergistically with the accumulation of osmotic adjustment substances in the drought response. In addition, root MDA was positively correlated with APX, Ss, Sp, and Pro, with correlation coefficients of 0.45, 0.68, 0.59, and 0.64, respectively, suggesting that the increase in membrane lipid peroxidation in roots under drought stress may be accompanied by a simultaneous enhancement of defense responses.

### 2.10. Principal Component Analysis

**Figure 10:**
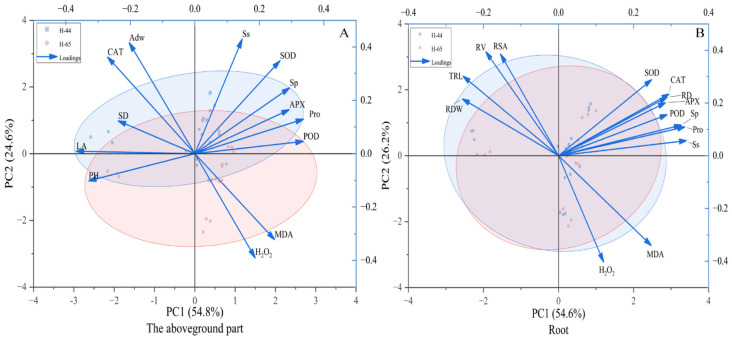
Principal component analysis of morphological and physiological indicators in soybean shoots and roots under drought stress. The PCA was performed using data from both soybean genotypes, all DPC treatments, and all biological replicates at 12 days after PEG-6000 treatment. (**A**) PCA of shoot indicators; (**B**) PCA of root indicators. PH, plant height; SD, stem diameter; LA, leaf area; Adw, aboveground dry weight; RDW, root dry weight; RV, root volume; RSA, root surface area; TRL, total root length; MDA, malondialdehyde; H_2_O_2_, hydrogen peroxide; CAT, catalase; SOD, superoxide dismutase; POD, peroxidase; APX, ascorbate peroxidase; Ss, soluble sugar; Pro, proline; Sp, soluble protein.

To further explore the differences and intrinsic relationships in the morphological and physiological responses of the shoots and roots of the two soybean varieties to DPC application under drought stress, principal component analysis (PCA) was performed on the shoot and root indicators at 12 d of treatment (Figure 10).

The PCA results for shoot indicators showed that PC1 and PC2 explained 54.8% and 24.6% of the total variance, respectively, with a cumulative contribution rate of 79.4%, adequately representing the primary information of the original dataset. PC1 mainly reflected the stress physiology of the plants; antioxidant enzymes (SOD, CAT, APX, POD) and osmotic adjustment substances (Sp, Pro) clustered in the first quadrant, showing a positive correlation with PC1, whereas morphological indicators (LA, PH, ST) concentrated in the second and third quadrants, showing a negative correlation with PC1. PC2 was primarily associated with oxidative damage, with MDA and H_2_O_2_ clustering in the fourth quadrant and correlating negatively with PC2. Furthermore, the score distribution of H-44 mainly shifted toward the positive direction of PC2, aligning closely with the loading directions of indicators such as aboveground dry weight (Adw), SOD activity, and Ss content. This indicates that under DPC regulation, the shoots of H-44 tended to resist drought by maintaining higher carbohydrate accumulation and antioxidant capacity. Conversely, H-65 mainly shifted toward the negative direction of PC2, showing a closer association with PH, LA, and oxidative damage indicators (MDA, H_2_O_2_), suggesting that the shoots of H-65 might have experienced relatively more pronounced membrane lipid peroxidation stress under DPC regulation.

The response pattern of roots differed from that of shoots. For root indicators, PC1 and PC2 explained 54.6% and 26.2% of the variance, respectively, with a cumulative contribution rate of 80.8%. The loading plot showed that root morphological indicators (RDW, TRL, RV, RSA) clustered on the negative side of PC1 and the positive side of PC2, whereas antioxidant enzymes (SOD, CAT, APX, POD) and osmotic adjustment substances (Sp, Pro, Ss) were mainly distributed on the positive side of PC1. The distribution pattern of MDA and H_2_O_2_ was similar to that in shoots, also locating in the fourth quadrant and correlating negatively with PC2. In addition, the confidence ellipses of H-44 and H-65 highly overlapped in the root PCA, indicating that DPC induced similar overall responses in the root morphology and physiological systems of both soybean varieties.

## 3. Discussion

Under drought stress, plants typically alter their morphology to reduce transpiration loss and enhance water acquisition [20]. The results of this study showed that drought stress significantly inhibited the increases in plant height and leaf area of the two soybean varieties. Following DPC application, the inhibition of plant height and leaf area was further exacerbated, whereas stem diameter increased significantly. This indicates that DPC, as a plant growth retardant, primarily limits longitudinal cell elongation and promotes lateral development by inhibiting gibberellin synthesis. Although this effect leads to reduced plant height and leaf area, it effectively decreases the transpiration area of the plant while strengthening the stem [17,22]. In contrast to the inhibitory effect on shoots, DPC significantly promoted the morphological development of soybean roots under drought stress. This study demonstrated that appropriate concentrations of DPC significantly restored the total root length, root surface area, root volume, and average diameter in both varieties, with the optimal DPC concentration varying between varieties with different drought tolerances (D200 for H-44 and D275 for H-65). Notably, although H-44 is considered the drought-tolerant genotype, it did not show the highest values for all morphological traits in the present study. This may be because drought tolerance is not determined solely by greater absolute growth, but is also associated with the ability of plants to maintain physiological stability and coordinate growth-defense trade-offs under stress. Under seedling-stage PEG-induced osmotic stress, H-44 may have adopted a relatively conservative growth strategy, allocating more resources to stress adaptation rather than rapid morphological expansion. This synergy between moderate shoot growth retardation and enhanced underground root development collectively improved the water uptake and retention capacity of soybean under drought stress. However, as the DPC concentration continued to increase, its alleviating effect on drought stress exhibited an initial increase followed by a decrease. This may be because excessively high DPC concentrations severely inhibit shoot growth, leading to a decline in the plant’s photosynthetic light-interception capacity, thereby weakening its overall ability to cope with drought [23].

Biomass accumulation and allocation are the ultimate material reflections of plant responses to environmental stress. Numerous studies have shown that drought stress significantly decreases total plant biomass and drives more assimilates to be translocated to the roots, thereby improving the overall coordination between water-absorbing and water-consuming organs [24,25,26]. However, prolonged allocation imbalance can restrict the development of photosynthetic organs. Wang et al. [23] reported that mepiquat chloride could significantly promote dry matter accumulation in the shoots and roots of soybean under drought stress. The present study found that foliar application of appropriate DPC concentrations significantly increased the leaf and stem dry weights of drought-stressed soybean while maintaining a high root biomass investment, leading to a significant recovery in the shoot dry weight/total dry weight (SDW/TDW) ratio compared with the DS treatment. This indicates that DPC can optimize carbon allocation under drought conditions, effectively alleviating the excessive inhibition of shoot biomass accumulation while maintaining root development, thus preserving a dynamic balance in inter-organ development. Furthermore, a root–shoot allometric growth model was used to quantify the relationship between biomasses, where the allometric scaling exponent (slope b) reflects the plant’s strategy in trading off aboveground and underground resource allocation [27]. The results revealed that under normal conditions, plants maintained a specific synchronized root–shoot growth pattern; drought disrupted this balance, causing a significant decrease in slope b. Appropriate DPC concentrations effectively restored the allometric scaling slopes of both varieties, suggesting that DPC can partially reconstruct the coordination between the roots and canopy of drought-stressed plants. However, when the DPC concentration was excessively high (D500), the extreme inhibition of shoots led to an abnormal shift in the allometric scaling slope, further confirming that DPC-mediated drought-resistant morphological regulation exhibits pronounced concentration and genotype specificity.

Drought stress disrupts the dynamic equilibrium between reactive oxygen species (ROS) production and scavenging in plant cells, leading to excessive ROS accumulation and subsequently triggering severe membrane lipid peroxidation [28,29]. In this study, as the duration of drought stress was extended to 12 d, the H_2_O_2_ and MDA contents in both soybean leaves and roots increased significantly. Notably, roots, as the primary organs directly perceiving the water-deficit environment, exhibited more severe oxidative damage. This may be associated with the direct exposure of roots to the PEG-induced low-water-potential environment. Under osmotic stress, roots first perceive a rapid decline in external water potential, resulting in restricted water uptake, disturbed cellular hydration status, and reduced membrane structural stability [30]. These changes may interfere with mitochondrial electron transport and plasma membrane-associated redox processes, thereby accelerating ROS production. In addition, roots must maintain water absorption, ion balance, and basic metabolic activities under stress conditions, which may further increase their energy demand and redox pressure [31]. Therefore, compared with leaves, roots experience an earlier and more direct imbalance between ROS production and scavenging, ultimately leading to higher H_2_O_2_ accumulation and more pronounced membrane lipid peroxidation. To mitigate drought-induced damage, plants activate antioxidant enzyme systems to scavenge excess ROS [32]. This study found that drought stress significantly increased the activities of SOD, POD, and APX in the leaves of both varieties, whereas CAT activity decreased. Application of appropriate DPC concentrations not only further enhanced SOD, POD, and APX activities but also significantly alleviated the drought-induced inhibition of CAT activity, with the optimal concentration varying between varieties (D200 for H-44 and D275 for H-65). Furthermore, DPC significantly reduced H_2_O_2_ and MDA contents in both organs under drought stress. These results indicate that DPC does not act merely locally in a single organ but systematically activates the overall antioxidant defense network of the plant, thereby efficiently scavenging ROS and protecting cell membrane integrity. In addition to the antioxidant enzyme system, osmotic adjustment is another key mechanism by which plants cope with drought stress and maintain cell turgor and water uptake. As important small-molecule osmolytes, Pro, Ss, and Sp can effectively lower cellular osmotic potential and protect the structures of biological macromolecules [33,34]. The results of this study showed that drought stress significantly increased the contents of Pro, Ss, and Sp in leaves and roots, while appropriate DPC concentrations further amplified this accumulation effect, with the accumulation of osmotic adjustment substances in roots being more sensitive to DPC application. However, when the DPC concentration was excessively high, antioxidant enzyme activities and osmotic adjustment substance contents generally decreased in the leaves and roots of both varieties, accompanied by renewed increases in MDA and H_2_O_2_ contents. This suggests that excessive DPC concentrations exceeded the tolerance threshold of the plants, disrupted normal physiological metabolism, and consequently induced secondary oxidative stress [35]. In addition, PEG-6000 was used in this study to simulate osmotic stress. This approach offers the advantages of relatively controllable stress intensity and good reproducibility; however, PEG treatment cannot fully replicate water movement, root–soil interactions, or aeration conditions under soil drought. Therefore, the conclusions of this study should mainly be confined to the seedling stage under controlled osmotic stress conditions. The appropriate dosage, response stability, and yield effects of DPC under field soil drought conditions still require further validation through pot experiments with soil culture and field trials.

## 4. Materials and Methods

### 4.1. Plant Materials

The soybean cultivars used in this experiment were Heinong 44 (H-44), characterized as drought-tolerant, and Heinong 65 (H-65), characterized as drought-sensitive. Seeds of both cultivars were provided by the Heilongjiang Academy of Agricultural Sciences. H-44 has a growth duration of approximately 118 days and requires an accumulated active temperature of approximately 2400 °C, whereas H-65 has a growth duration of approximately 115 days and requires an accumulated active temperature of approximately 2350 °C. The similar growth durations of the two cultivars help reduce the influence of differences in developmental progress on the comparison of drought responses. The plant growth regulator used in this experiment was mepiquat chloride, also known as 1,1-dimethylpiperidinium chloride (DPC). The DPC reagent was a soluble powder with a purity of ≥98% and was supplied by Hebei Guoxin Nuonong Biotechnology Co., Ltd., Cangzhou, China.

### 4.2. Experimental Design and Treatments

The experiment was conducted in 2025 in a glass rain-shelter greenhouse at the experimental base of Northeast Agricultural University, China, to investigate the alleviating effects of different DPC concentrations on drought stress in soybean. The rain-shelter greenhouse was used mainly to exclude natural rainfall and ensure consistency among water treatments. Temperature, relative humidity, and light conditions were not artificially controlled during the experiment. A pot experiment with sand culture was performed using plastic pots with drainage holes at the bottom. Each pot was 30 cm in height and 28 cm in diameter. The bottom of each pot was lined with gauze mesh and then filled with pre-washed river sand. Eight soybean seeds with full grains, uniform size, and no visible symptoms of disease or insect damage were sown in each pot. When the opposite unifoliate leaves were fully expanded, seedlings were thinned, and three uniform plants were retained in each pot. From sowing to the full expansion of the opposite unifoliate leaves, each pot was irrigated daily with 500 mL of water. After thinning, each pot was irrigated once daily with 500 mL of Hoagland nutrient solution to ensure normal nutrient supply. The Hoagland nutrient solution was prepared according to the method described by Dong et al. [36], and its detailed composition is shown in Table 2.

Treatments were initiated when the two trifoliate leaves were fully expanded, corresponding to the V2 stage. This stage is a critical period of vegetative growth during the soybean seedling stage, is relatively sensitive to water deficit, and is suitable for evaluating the effects of drought stress on early growth and root establishment. Six treatments were established: control treatment (CK), drought stress treatment (DS), and four DPC treatments at concentrations of 125, 200, 275, and 500 mg/L, designated as D125, D200, D275, and D500, respectively. The DPC concentration range was determined based on preliminary experiments conducted in our laboratory to cover low, medium, and high concentration levels [23]. Each treatment included 24 pots, with a total of 144 pots. During treatment application, plants in the CK and DS groups were sprayed with an equal volume of water, whereas plants in the DPC treatment groups were foliar-sprayed with the corresponding DPC solutions until the leaf surface was completely wetted without dripping (each pot received 75 mL of the corresponding solution). A second foliar application was performed 3 days after the first application. Drought stress was initiated after the second foliar application. Plants in the CK treatment were irrigated daily with 500 mL of Hoagland nutrient solution, whereas plants in the DS and DPC treatments were irrigated daily with 500 mL of Hoagland nutrient solution containing 10% PEG-6000 to simulate drought stress. The PEG-6000 concentration was selected based on previous studies in our laboratory, and the corresponding water potential was −0.20 MPa [37].

Plant sampling was performed between 08:00 and 09:00 on days 6 and 12 after the initiation of PEG-6000 treatment. For each treatment, six pots were randomly selected at each sampling time, representing six independent biological replicates. Among them, three pots were used for physiological measurements. The second and third fully expanded trifoliate leaves from the shoot apex, together with root tip samples, were collected for physiological assays. Specifically, root tip segments were excised from cleaned roots, pooled within each pot, immediately frozen in liquid nitrogen, and stored at −80 °C for subsequent physiological assays. The other three pots were used for shoot morphological measurements, and their roots were washed for scanning and root morphological analysis. For both physiological and morphological measurements, the pot was considered the experimental unit, and the three seedlings within each pot were pooled as one biological replicate.

### 4.3. Measurement Items and Methods

#### 4.3.1. Determination of Morphological Traits and Biomass

At each sampling time, the shoots were cut at the cotyledonary scar, and plant height was measured manually with a meter ruler as the distance from the cotyledonary node to the apical growing point. After sampling, roots were rinsed thoroughly with water to eliminate attached soil or substrate particles. The cleaned root systems were placed in a transparent acrylic tray filled with deionized water, and individual root segments were carefully arranged with forceps to ensure clear separation during scanning. Digital root images were obtained using a Phantom 9900XL scanner (Microtek, Shanghai, China) at a resolution of 400 dpi and saved in TIFF format. Root morphological parameters, including total root length, root surface area, average root diameter, and root volume, were extracted from the images using WinRHIZO v2019 software (Regent Instruments Inc., Québec, QC, Canada).

Leaf area was determined after all leaves had been detached from each plant. The leaves were spread flat on a white plate and covered with transparent film to prevent curling. The total leaf area per plant was then measured using a Yaxin-1241 portable leaf area meter (Beijing Yaxinliyi Technology Co., Ltd., Beijing, China). For dry matter measurement, leaves, stems, petioles, and roots were separated before drying. Each sample was placed in a pre-weighed aluminum box, treated at 105 °C for 30 min to terminate metabolic activity, and then dried at 65 °C until a stable dry mass was reached. Leaf dry weight, stem dry weight, petiole dry weight, and root dry weight were recorded separately after cooling, and shoot dry weight was calculated as the sum of leaf, stem, and petiole dry weights.

#### 4.3.2. Determination of MDA and H_2_O_2_ Contents

The concentration of malondialdehyde (MDA) was determined by the thiobarbituric acid (TBA) reaction following the method of [38]. Hydrogen peroxide (H_2_O_2_) content was measured using the potassium iodide (KI) assay as described by [39].

#### 4.3.3. Determination of Antioxidant Enzyme Activities

To extract antioxidant enzymes, fresh leaf tissue (0.1 g) was homogenized in 1 mL of corresponding ice-cold extraction buffers. The activities of superoxide dismutase (SOD), peroxidase (POD), and ascorbate peroxidase (APX) were determined following the established protocols described by [40]. Briefly, SOD activity was assayed based on the photochemical reduction in nitroblue tetrazolium (NBT) at 560 nm; POD activity was measured via the guaiacol oxidation method at 470 nm; and APX activity was determined by tracking the decrease in absorbance at 290 nm. Catalase (CAT) activity was assessed according to the method of [41] by monitoring the decomposition of H_2_O_2_ at 240 nm. All enzyme activities were calculated and expressed as units per gram of fresh weight (U g^−1^ FW).

#### 4.3.4. Determination of Osmotic Adjustment Substances

The concentrations of key osmotic adjustment substances, including proline (Pro), soluble protein (Sp), and soluble sugars (Ss), were determined spectrophotometrically. Proline was extracted from fresh tissue (0.1 g) and quantified using the acid ninhydrin assay at 520 nm according to [42]. Soluble protein content was measured using the Coomassie Brilliant Blue G-250 dye-binding method at 595 nm as described by [43], with bovine serum albumin (BSA) serving as the standard. Soluble sugar concentration was analyzed from desiccated samples (0.1 g) using the anthrone colorimetric assay at 625 nm following the protocol of [44]. Pro and Sp contents were expressed on a fresh weight basis (μg g^−1^ FW), whereas Ss content was expressed on a dry weight basis (mg g^−1^ DW).

### 4.4. Data Processing and Statistical Analysis

All experimental data were organized and tabulated using Microsoft Office Excel 2010. Statistical analyses were performed using IBM SPSS Statistics version 21.0 (IBM Corp., Armonk, NY, USA), with the significance level set at α = 0.05. Differences among treatments were analyzed by one-way analysis of variance (ANOVA) followed by Duncan’s multiple range test. Before ANOVA, data normality was assessed using the Shapiro–Wilk test (*p* > 0.05), and homogeneity of variance was evaluated using Levene’s test (*p* > 0.05). Statistical graphs, correlation heatmaps, and principal component analysis (PCA) plots were generated using Origin 9 (OriginLab Corp., Northampton, MA, USA).

To decipher biomass partitioning strategies across the diverse treatments, an allometric growth framework was adopted. This biological scaling is fundamentally captured by the power-law equation:

Y = aX^b^

For robust linear regression modeling, both sides of the equation underwent a base-10 logarithmic transformation, yielding the standardized linear form:

log_10_(Y) = log_10_(a) + b × log_10_(X)

Within this context, the variables X and Y denote the dry masses of the shoot and root, respectively; a functions as the regression intercept, while b serves as the allometric scaling exponent. The allometric growth regression was fitted using the combined dataset from both sampling times, namely 6 and 12 days after PEG-6000 treatment. For each soybean genotype and DPC treatment, all biological replicates from both sampling times were included in the regression analysis. Origin 9 facilitated both the dataset transformation and the subsequent linear fitting procedures. From these treatment-specific regression outputs, the scaling slope (b), the transformed intercept (log_10_a), and the explanatory power (R^2^) were extracted. The data used for PCA, correlation analysis, and allometric growth regression are provided in the Supplementary Materials.

## 5. Conclusions

In conclusion, DPC at appropriate concentrations effectively alleviated drought-induced inhibition of soybean growth. DPC moderately restricted shoot expansion while significantly promoting root growth, thereby modifying the root–shoot allometric trajectory under drought stress and optimizing dry matter allocation. Meanwhile, DPC coordinately regulated antioxidant enzyme activities and osmotic adjustment capacity in leaves and roots, reduced the accumulation of H_2_O_2_ and MDA, and consequently maintained cellular homeostasis at the whole-plant level. This regulatory effect showed marked genotype specificity and concentration dependence, with the optimal alleviation observed at 200 mg/L DPC for the drought-tolerant cultivar H-44 and 275 mg/L DPC for the drought-sensitive cultivar H-65. However, the stress-regulatory effect of DPC is subject to a strict concentration threshold. Therefore, the application dosage should be carefully controlled in practical use, as excessively high foliar concentrations (≥500 mg/L) may induce secondary stress due to excessive growth inhibition.

## Figures and Tables

**Table 1:** Allometric relationship between root and shoot biomass of soybean under drought stress and DPC regulation.

	Intercept		Slope		Pearson’s r		R2	
Treatment	H-44	H-65	H-44	H-65	H-44	H-65	H-44	H-65
CK	−0.95 ± 0.19	−0.39 ± 0.08	1.45 ± 0.19	0.87 ± 0.09	0.97	0.98	0.94	0.96
DS	0.15 ± 0.07	−0.07 ± 0.09	0.32 ± 0.08	0.58 ± 0.11	0.9	0.94	0.81	0.88
D125	0.02 ± 0.10	−0.03 ± 0.05	0.48 ± 0.11	0.54 ± 0.06	0.91	0.98	0.83	0.96
D200	−0.01 ± 0.07	−0.05 ± 0.09	0.51 ± 0.08	0.56 ± 0.10	0.96	0.94	0.92	0.89
D275	0.01 ± 0.08	−0.17 ± 0.11	0.47 ± 0.08	0.72 ± 0.12	0.94	0.95	0.89	0.9
D500	0.16 ± 0.18	0.02 ± 0.13	0.84 ± 0.21	1.02 ± 0.15	0.9	0.96	0.81	0.92

Note: The allometric growth relationship is expressed as Log(Root) = a + b × Log(Shoot), where b is the allometric slope, and a is the intercept. R^2^ represents the coefficient of determination. CK: Control; DS: Drought stress; D125–D500: Drought stress with 125–500 mg/L DPC application.

**Table 2:** Composition of the Hoagland nutrient solution.

Inorganic Salts	Concentration (mg/L)	Inorganic Salts	Concentration (mg/L)
MgSO_4_	240.00	CuSO_4_·5H_2_O	0.08
KH_2_PO_4_	136.00	ZnSO_4_·7H_2_O	0.22
NH_4_NO_3_	142.86	MnCl_2_·4H_2_O	4.90
CaCl_2_	220.00	H_3_BO_3_	2.86
Na_2_MoO_4_·H_2_O	0.03	Fe-EDTA	*

* Note: Fe-EDTA stock solution was prepared by dissolving 5.57 g FeSO_4_·7H_2_O and 7.45 g Na_2_EDTA separately and adjusting each solution to 1 L. For each liter of nutrient solution, 1 mL of stock solution was added.

## Data Availability

The original data presented in this study are included in the article.

## Supplementary Materials

The following supporting information can be downloaded at: https://www.mdpi.com/article/10.3390/plants15132031/s1. Table S1: Allometric regression analysis data of the two cultivars; Table S2: Correlation analysis data of leaf and root traits; Table S3: Principal component analysis (PCA) data of leaf and root traits.
